# Household income during childhood and risk of youth suicide attempts and deaths: a register-based study from Finland

**DOI:** 10.1007/s00127-026-03140-1

**Published:** 2026-06-15

**Authors:** Veera Nieminen, Kimmo Suokas, Reija Autio, Christian Hakulinen, Kari Aaltonen, Sami Pirkola

**Affiliations:** 1 Faculty of Social Sciences, Health Sciences, Tampere University, Arvo Ylpön katu 34, 33250 Tampere, Finland; 2 Department of Psychology, Faculty of Medicine, University of Helsinki, Helsinki, Finland; 3 Finnish Institute for Health and Welfare, Helsinki, Finland; 4 Tampere University Central Hospital, Tampere, Finland

**Keywords:** Suicide, Suicide attempt, Childhood income, Risk factors, Adolescence, Young adulthood

## Abstract

**Purpose:**

Socioeconomic inequalities during childhood are related to a variety of adverse mental health outcomes. However, youth suicide deaths have rarely been studied in this respect. We examined whether household income during childhood is associated with the risk of youth suicide attempts and deaths.

**Methods:**

In this register-based cohort study all individuals born in Finland between 1991 and 2005 were followed from the age of 15 to either suicide or suicide attempt, death by other causes, emigration, or the end of the study period (31st December 2020). Household income was measured throughout childhood with two commonly used measures, cumulative income and income trajectories, and at specific ages (5, 10, and 15). Penalized splines and Cox regression models estimated the associations between household income and suicide attempts and deaths. We selected additionally adjusted models with a best-fitting set of cofactors for both outcomes. Additionally, we reported income-specific cumulative risks.

**Results:**

During the follow-up period of 6.8 million person-years (*n* = 886,748), 9,811 first suicide attempts (60.9% females) and 964 suicides (28.5% females) occurred. Lower household income was associated with an increased risk of suicide attempts after adjusting for cofactors including parental mental disorders, suicide, education, urbanicity, birth origin and age. However, household income was not associated with suicide deaths when parental mental disorders were considered.

**Conclusion:**

Low income during childhood is associated with youth suicide attempts, but not with suicide deaths. Dying from suicide is a rare and extreme event, and treating non-lethal suicidal acts as a proxy for suicide requires caution.

**Supplementary Information:**

The online version contains supplementary material available at https://doi.org/10.1007/s00127-026-03140-1.

## Introduction

Suicide is the third leading cause of death globally for people aged 10–29 years and a significant contributor to years of life lost [1–3]. These deaths have severe consequences, as the young individual loses their life, and it is estimated that each death also affects an average of 135 other people [4]. Non-fatal suicidal behavior, including suicide attempts, peaks during late adolescence and early adulthood [1]. Suicide mortality increases most rapidly during adolescence, but peaks in older age groups [5]. In a recent meta-analysis, the global prevalence of suicide attempts among youths under 22 years old ranges from 4.6% to 15.8% [6]. The global suicide mortality rate among under 20-year-old adolescents has been estimated to be 3.77/100,000 people [7], and has shown an overall decline during the past three decades [2]. However, the cross-national variability in youth suicide rates and trends is high. Recently, youth suicide mortality in Finland has been estimated to remain above the average in international comparison [8].

Suicide attempts and deaths are often discussed together in the context of self-harm and suicidal behavior. They are necessarily interlinked, but knowledge regarding the extent to which the risk factors for non-lethal suicide attempts and deaths are distinct and overlapping, is still limited. The majority of young individuals who die by suicide die on their first attempt [9]. In turn, most of those who attempt suicide survive, although the risk of suicide remains elevated [10]. Depression and other mental disorders, hopelessness, and impulsive-aggressive traits are reported to be associated with increased risk of both suicide death and attempts during adolescence and early adulthood [11, 12]. Differences have been reported, for example in relation to sociodemographic factors: suicide deaths are more common among males, and attempts among females and younger people [11–13].

Suicidal behavior is also affected by societal and socioeconomic factors [1]. Of the different indicators of socioeconomic position, income reflects the resources readily available in the family, and it correlates with other socioeconomic measures, such as housing, neighborhood, and education [14]. In previous register-based studies, low parental income was associated with increased risk of suicide attempts [15, 16]. Although low parental income has also been associated with an increased risk of youth suicide [17], there is evidence to the contrary, with studies suggesting that higher parental income is associated with an increased suicide risk [18, 19]. To the best of our knowledge, there appears to be no recent population-based studies examining how parental income is associated with both youth suicide attempts and suicide. Furthermore, previous studies have mainly focused on income at a specific point in time, whereas the association of household income and its changes throughout childhood with the risk of suicide has not been examined. In summary, there remains a notable scarcity of studies on adolescents with suicide death as an outcome [20], as well as those that examine both suicide attempts and deaths in same population.

In this study, using nationwide registry data, we examined whether household income during childhood is associated with both youth suicide attempts and deaths.

## Methods

### Study population and data sources

We linked demographic and socioeconomic data from Statistics Finland with information from the Causes of Death Register and Finnish Care Register for Health Care (FCRHC). FCRHC, maintained by the Finnish Institute for Health and Welfare, covers information on inpatient hospital admissions in Finland since 1970, outpatient care since 1998, and primary care since 2011 [21]. Data linkage utilized personal identity codes, which we also used to link parental information with study subjects. Data were linked with permission from Statistics Finland (TK–53–1696-16) and the Finnish Institute of Health and Welfare. Informed consent is not required in register-based studies in Finland. The study followed the guidelines of the Declaration of Helsinki. A cohort of all individuals born in Finland between 1991 and 2005 (*n* = 886,748) was followed from their 15th birthday to either (1) first suicide attempt, death, emigration, or the end of the study period (31st December 2020), or (2) suicide death, death for other reason, emigration, or the end of the study period, whichever came first. The inclusion criterion of being born in Finland was related to the incompleteness of income data among parents who have migrated from other countries.

### Suicide attempt

In this study, we define suicide attempt as cases in which the person has been in contact with a health care provides, and a clinician has assessed that the person has intentionally harmed themselves using a method that could also lead to death. Utilizing FCRHC, we collected all the somatic or psychiatric inpatient and outpatient visits from secondary care and all the outpatient visits from primary care due to ICD-10 codes (X60-X84) for the suicide attempts [22]. Only non-fatal attempts were considered as suicide attempts, meaning that those who died of suicide at their first attempt were not included among those who attempted suicide.

### Suicide death

Information on suicide was retrieved from the Cause of Death Register, based on ICD-10 codes X60–X84 and Y87.0, which describe suicide and intentional self-harm [22].

### Household income measures

Household income measures were based on the equivalized disposable income for each household dwelling unit, which was calculated with the OECD-modified Equivalence Scale [23]. This income measure is adjusted for inflation and the size of the household. Based on the equivalized disposable income, the study subjects were assigned to percentiles (1-100) and quintiles (1–5), relative to the whole household income distribution of Finland. The income measures were obtained for each year in the study. We employed both income measures that have been primarily used in previous studies, including cumulative measures [16, 24] and trajectories [25]. Using calculated income quintiles, we formed a cumulative household income scale by summing income quintiles measured at ages 0, 5, 10 and 15. This scale ranges from 4 to 20, with the value 4 indicating the lowest quintile at all measurement points, and the value 20 the highest quintile at all measurement points. For example, if the household’s income is in the lowest quintile at all measurement points (at ages 0, 5, 10, and 15), the value of the measure is 4 (1 + 1+1 + 1). In turn, if the household’s income is in the highest quintile at all measurement points, the value is 20 (5 + 5+5 + 5). In addition, we used household income percentiles between ages 1 to 15 to calculate household income trajectories, using the *latrend* package in R [26]. The number of clusters was selected using log likelihood and Bayesian information criteria (BIC) comparison. These household income trajectories utilize the household income level at each age and can detect changes in household income levels.

### Cofactors

We considered several factors that could function as common causes for both household income up to child’s age 15, and suicide attempt or death at ages 15–30. Since the association between income and health can vary substantially in its strength and shape by level of education [27], we included parental education based on the highest completed degree, categorized as basic, upper secondary, short-cycle tertiary, lower degree tertiary and higher degree tertiary education. Given that Finnish education system is inclusive and dropping out is uncommon, we coded lacking information of completed degree (0.2% for mothers and 1.8% for fathers) to the lowest educational category (basic education) [28]. To assess parental mental disorder, we used FCRHC register to detect whether either parent had a record of any mental disorder by the time the child turned 15. Parental mental disorders were coded as “neither”, “mother”, “father”, and “both”. Parental mental disorder could affect work ability and income [29] and, on the other hand, the child’s risk for suicide attempt or death [15, 17, 30]. Parental suicide before the child’s age 15 was coded as “yes” or “no”. Parent’s death of suicide is one of the strongest risk factors for offspring’s suicide [31]. Since the death of a parent also affects the family’s income level, we decided to include this as a confounding factor, even though the equivalized household income itself considers the number of adults and children living in the household. A three-level household urbanicity classification (urban, semi-urban, rural) was based on the status at child’s age 1. 81% of the study subjects were in the same category at ages 1 and 15. Additionally, we included parental birth origin (Finnish or other), and parental age. Missing values in parental age and urbanicity were coded as a separate category to ensure that observations with missing data would also be included in the analyses.

### Statistical analysis

This study employs three approaches in statistical modeling: penalized splines models, minimally and additionally adjusted Cox regression models, and cumulative risks. We calculated hazard ratios (HRs) and 95% confidence intervals (CIs) with stratified Cox proportional hazard models with a robust standard error estimator, using *survival* package in R [32]. We utilized penalized splines models with 5 degrees of freedom to flexibly model nonlinear associations between cumulative income quintiles and suicide attempts and suicide, and at the same time avoid overfitting. Cumulative income quintile was included as a categorical explanatory variable and the median of the cumulative income quintiles (10) served as a reference. In Cox regression models, we included three different income measures as explanatory variables: (1) cumulative income quintile as a continuous variable to assess the overall association between income and suicide attempts and deaths, (2) income trajectories as a categorical variable, with the reference category “middle”, and (3) household income quintile in a specific point of time (at child’s age 5, 10, and 15), with the reference value of 3 (middle). Minimally adjusted Cox regression models included the income exposure and were stratified with categorized birth year. In additionally adjusted models for suicide attempts, we included also parental age, urbanicity, birth origin, education and mental disorder, and for suicide, parental mental disorder. Best-fitting sets of covariates were selected for the models for each outcome based on BIC comparisons. The assumption of proportional hazards was assessed with Schoenfeld’s residuals. We used stratification to allow varying baseline hazards on the categorized birth year, and to calculate a mutual HR for all levels of strata. In addition, cumulative risks up to 30 years were calculated with the Aalen-Johansen estimator. For suicide attempts, the competing risks included deaths and emigration, and for suicide deaths, deaths from other causes and emigration. The cumulative risk was estimated at the end of the follow-up. All models were created separately for suicide attempts and deaths, and due to gender differences in the outcomes, for both genders separately. Statistical analyses were conducted with R, version 2024.09.0.

## Results

Over the follow-up period of over 6.8 million person-years (*n* = 886,748; 48.8% females), 964 suicides (28.5% females) and 9,811 first suicide attempt cases (60.9% females) occurred. 145 study subjects who had a first suicide attempt after their 15th birthday, died of suicide before the end of the follow-up. The detailed characteristics of the study sample are presented in Table 1.

**Table 1:** Characteristics of subjects who had a first suicide attempt, died of suicide, and the whole study population

Characteristic	Suicide Attempt	Suicide Death	All
Total number	9,811	964	886,748
**Gender**			
Male	3,833 (39.1)	689 (71.5)	453,741 (51.2)
Female	5,978 (60.9)	275 (28.5)	433,004 (48.8)
**Birth year**			
1991–1995	4,809 (49.0)	632 (65.6)	320,841 (36.2)
1996–2000	3,499 (35.7)	268 (27.8)	287,105 (32.4)
2001–2005	1,503 (15.3)	64 (6.6)	278,802 (31.4)
**Income trajectory**			
Lowest	2,866 (29.2)	235 (24.4)	149,736 (16.9)
Second Lowest	2,309 (23.5)	237 (24.6)	174,068 (19.6)
Downward	758 (7.7)	76 (7.9)	76,552 (8.6)
Middle	1,648 (16.8)	204 (21.2)	171,697 (19.4)
Second Highest	1,027 (10.5)	120 (12.4)	173,815 (19.6)
Highest	422 (4.3)	41 (4.3)	96,427 (10.9)
Missing	781 (8.0)	51 (5.3)	44,453 (5.0)
**Parental background**			
Finnish (at least one parent)	9,668 (98.5)	959 (99.5)	869,422 (98.0)
Other	143 (1.5)	≤ 5 (0.5)	17,326 (2.0)
**Parental mental disorder**			
Neither	2,868 (29.2)	232 (24.1)	465,694 (52.5)
Mother	2,863 (29.2)	311 (32.3)	203,547 (23.0)
Father	1,500 (15.3)	114 (11.8)	117,524 (13.3)
Both	2,580 (26.3)	307 (31.8)	99,983 (11.3)
**Parental suicide**			
No	9,689 (98.8)	953 (98.9)	882,259 (99.5)
Yes	122 (1.2)	11 (1.1)	4,489 (0.5)
**Mother’s education**			
Basic	2,090 (21.3)	121 (12.6)	148,665 (16.8)
Upper secondary	4,590 (46.8)	428 (44.4)	343,782 (38.8)
Short-cycle tertiary	1,580 (16.1)	215 (22.3)	183,155 (20.7)
Lower degree tertiary	751 (7.7)	91 (9.4)	90,843 (10.2)
Higher degree tertiary	800 (8.2)	109 (11.3)	120,313 (13.6)
**Father’s education**			
Basic	3,283 (33.5)	227 (23.5)	223,541 (25.2)
Upper secondary	4,347 (44.3)	461 (47.8)	373,987 (42.2)
Short-cycle tertiary	884 (9.0)	99 (10.3)	109,541 (12.4)
Lower degree tertiary	583 (5.9)	79 (8.2)	76,312 (8.6)
Higher degree tertiary	714 (7.3)	98 (10.2)	103,367 (11.7)
**Mother’s age at birth**			
19y and younger	470 (4.8)	36 (3.7)	16,857 (1.9)
20-29y	5,289 (53.9)	457 (47.4)	421,620 (47.5)
30y and older	4,040 (41.2)	470 (48.8)	446,422 (50.3)
Missing	12 (0.1)	≤ 5 (0.1)	1,849 (0.2)
**Father’s age at birth**			
19y and younger	124 (1.3)	10 (1.0)	4,563 (0.5)
20-29y	3,928 (40.0)	311 (32.3)	298,403 (33.7)
30y and older	5,449 (55.5)	621 (64.4)	568,045 (64.1)
Missing	310 (3.2)	22 (2.3)	15,737 (1.8)
**Urbanicity**			
Urban	6,088 (62.1)	530 (55.0)	498,553 (56.2)
Semi-Urban	1,595 (16.3)	163 (16.9)	155,332 (17.5)
Rural	2,063 (21.0)	263 (27.3)	225,972 (25.5)
Missing	65 (0.7)	8 (0.8)	6,891 (0.8)

**Fig. 1:**
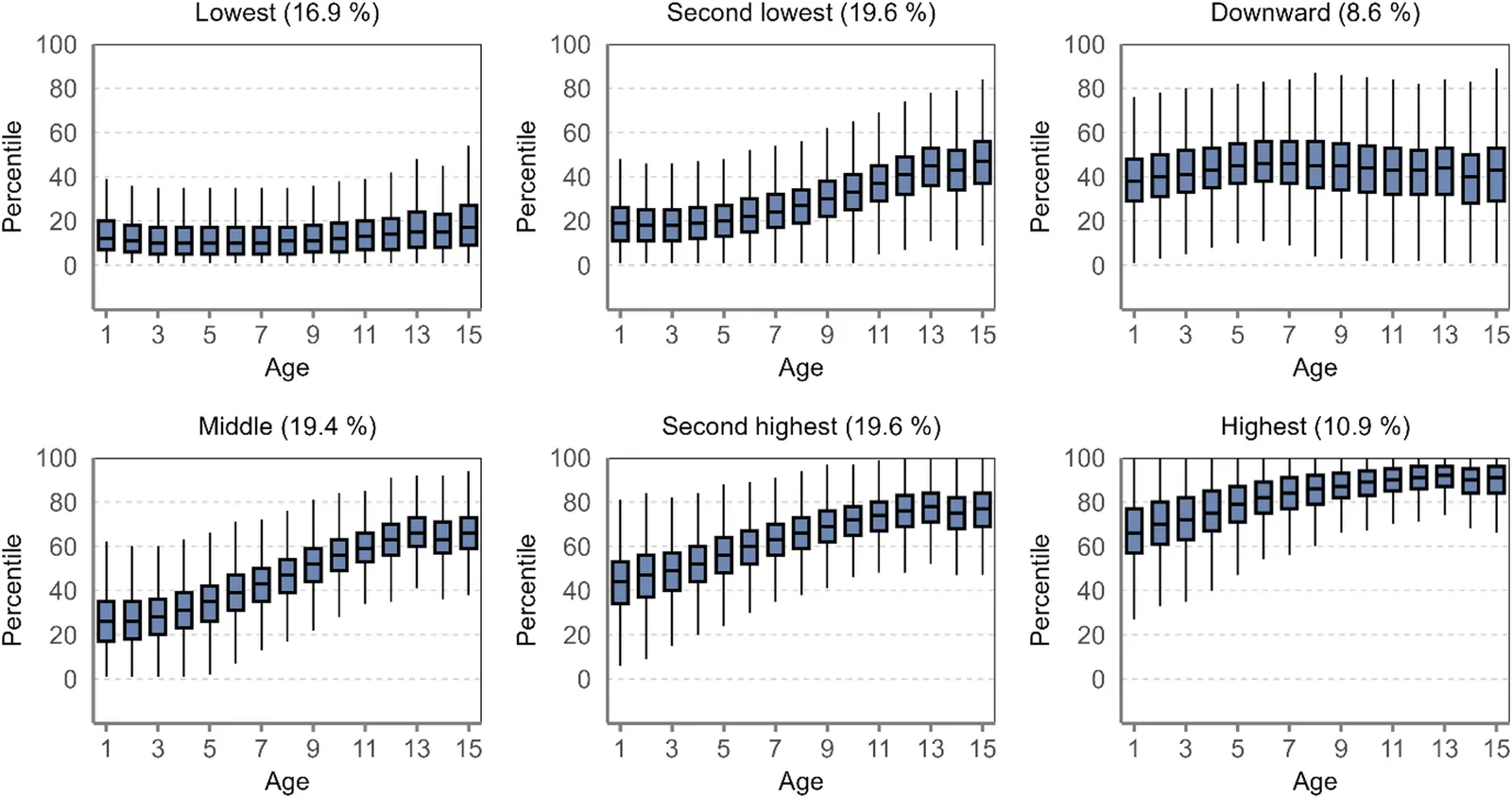
The income trajectories during childhood. Trajectories are based on parental income at child’s ages 1–15. 5.0% of the population had missing income information between ages 1–15

The income trajectories are presented in Fig. 1. Of the whole study population, 5.0% (44,453) had missing income data for at least one year between ages 1 and 15 and therefore were not assigned to any income trajectory but were analyzed as a separate group.

**Fig. 2:**
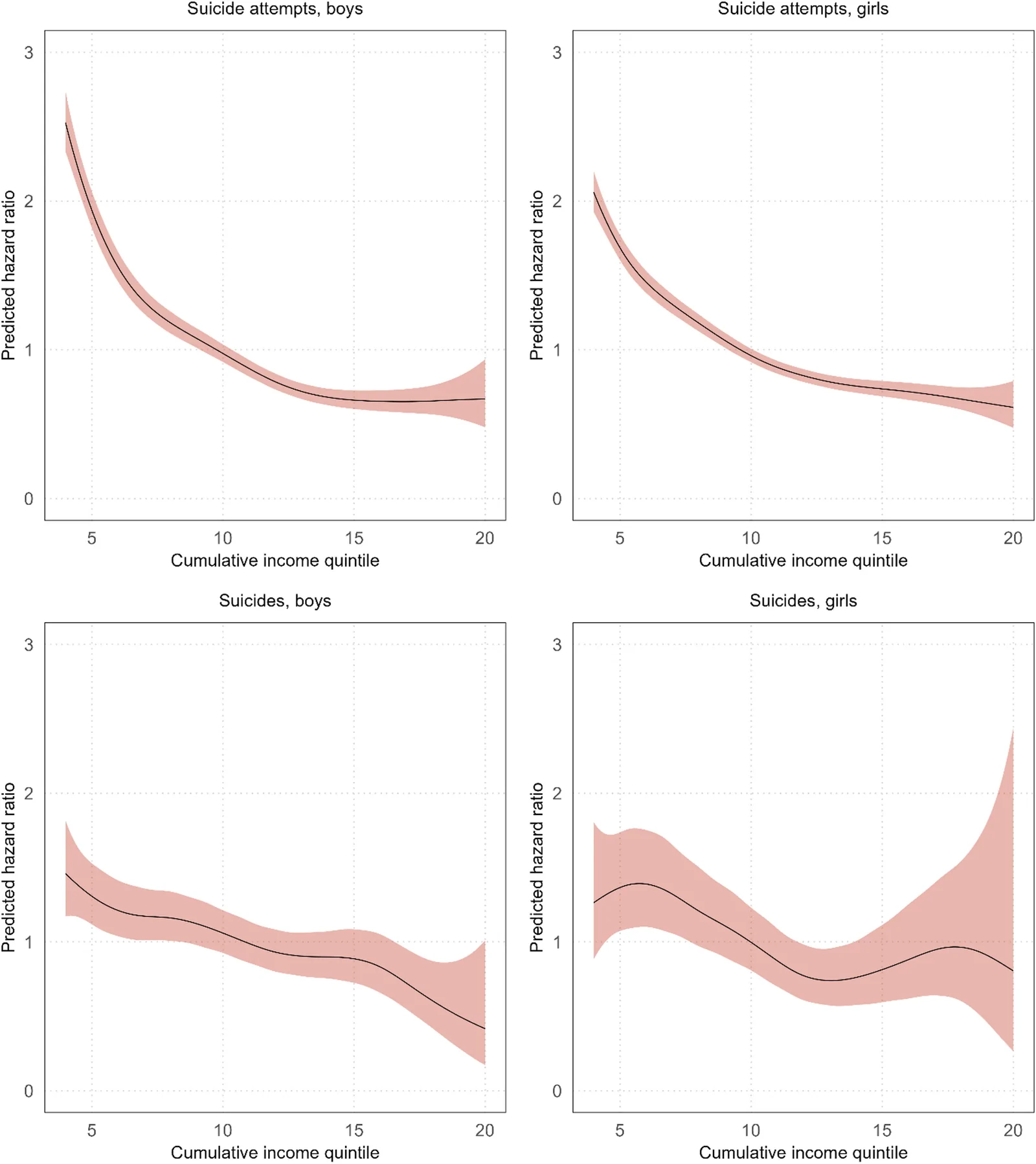
Crude associations of cumulative income quintiles with suicide and first suicide attempt, based on predicted hazard ratios from penalized splines models. The median of cumulative income quintiles (10) serves as the reference

For suicide attempts and household income measured with cumulative income quintiles, we observed an inverse non-linear relationship. Lower income was associated with higher predicted hazard ratio for both genders for suicide attempts. For suicide deaths, no such clear associations were observed. For boys, a slightly elevated risk of suicide in the lowest income groups was detected, and higher income groups showed a minor protective effect. For girls, these associations were not as evident. These results are shown in Fig. 2.

In minimally adjusted Cox regression models, one unit increase in cumulative income measure decreased the risk for both suicide attempts (for boys HR 0.89 [95% CI 0.88–0.89], for girls HR 0.90 [95% CI 0.90–0.91]) and suicide deaths (for boys HR 0.95 [95% CI 0.93–0.97], for girls HR 0.95 [95% CI 0.92–0.98]). After consideration of potential confounders, the association essentially remained in suicide attempts (for boys HR 0.94 [95% CI 0.93–0.95], for girls HR 0.95 [95% CI 0.94–0.95]), but was fully attenuated in suicides (for boys HR 1.00 [95% CI 0.98–1.02], for girls HR 1.00 [95% CI 0.97–1.04]).

The results of Cox regression models with income clusters (presented in Fig. 1) are shown in Table 2. For the minimally adjusted models of suicide attempts, the lowest and second lowest income trajectories were associated with higher risk for both genders. Furthermore, the highest and second highest income trajectories were associated with lower risk for both genders. The downward income trajectory was associated with increased risk only for girls. In the additionally adjusted models, these associations attenuated but remained significant among all the trajectories for both genders, except for the highest income trajectory among boys. For suicide, the lowest income cluster was associated with higher risk for both genders in the minimally adjusted models. For boys, high income was associated with lower risk. After adjusting for potential confounders, the associations were fully attenuated. 

**Table 2:** Associations of suicide attempts and deaths with childhood income trajectories. Results from stratified Cox regression models with minimal and additional adjustment. Minimally adjusted models were stratified with categorized birth year. The models examining suicides among boys and girls were stratified by year of birth and parental mental disorder. The models examining suicide attempts were stratified by year of birth as well as by parental age, parental education, parental mental disorder, parental suicide, parental birth origin (in Finland or abroad), and urbanicity

	Boys				Girls			
	Minimally adjusted		Additionally adjusted		Minimally adjusted		Additionally adjusted	
	HR	95% CI	HR	95% CI	HR	95% CI	HR	95% CI
**Suicide attempt**								
*Income trajectory*								
Lowest	**2.16**	**1.96–2.38**	**1.38**	**1.25–1.53**	**1.96**	**1.81–2.12**	**1.46**	**1.35–1.59**
Second lowest	**1.31**	**1.19–1.45**	**1.11**	**1.00–1.23**	**1.38**	**1.27–1.50**	**1.21**	**1.11–1.31**
Downward	1.13	0.98–1.30	1.05	0.90–1.21	**1.24**	**1.11–1.38**	**1.16**	**1.04–1.29**
Middle (ref.)	1		1		1		1	
Second highest	**0.64**	**0.56–0.73**	**0.74**	**0.65–0.85**	**0.75**	**0.68–0.83**	**0.85**	**0.77–0.94**
Highest	**0.68**	**0.57–0.81**	0.90	0.74–1.09	**0.62**	**0.54–0.71**	**0.83**	**0.72–0.96**
Missing	**2.23**	**1.95–2.54**	**1.45**	**1.25–1.68**	**1.89**	**1.69–2.11**	**1.49**	**1.31–1.68**
**Suicide death**								
*Income trajectory*								
Lowest	**1.28**	**1.03–1.60**	0.91	0.72–1.14	**1.47**	**1.03–2.10**	0.98	0.68–1.42
Second lowest	1.03	0.83–1.29	0.89	0.72–1.12	1.14	0.80–1.64	0.96	0.67–1.38
Downward	0.98	0.71–1.35	0.88	0.64–1.21	1.38	0.86–2.21	1.20	0.75–1.93
Middle (ref.)	1		1		1		1	
Second highest	0.81	0.63–1.06	0.90	0.69–1.17	0.72	0.46–1.13	0.83	0.53–1.30
Highest	**0.58**	**0.37–0.89**	0.73	0.47–1.33	1.01	0.59–1.75	1.39	0.80–2.44
Missing	1.09	0.76–1.56	0.84	0.55–1.28	1.16	0.65–2.10	0.90	0.50–1.62

The results of minimally and additionally adjusted Cox regression models with household income quintile at ages 5, 10, and 15 as an exposure, are presented in *Supplementary Fig. 1.*

The absolute risks of youth suicide attempts and deaths according to income trajectory are shown in Table 3. The absolute risk of suicide attempts in the lowest income trajectory was 2.70% [95% CI 2.52–2.89] for boys, and 3.48% [95% CI 3.29–3.68] for girls. The absolute risk of suicide in the lowest income trajectory was 0.42% [95% CI 0.36–0.50] for boys, and 0.17% [95% CI 0.13–0.23] for girls.

**Table 3:** The cumulative risk and 95% confidence intervals of suicide attempts and deaths, reported separately for both genders. The cumulative risks are derived from the Aalen-Johansen estimator, in which the only exposure was income trajectory, and the risk was estimated at the end of the follow-up. Competing risks included deaths from other causes and emigration

	Suicide attempt		Suicide death	
	Boys (%)	Girls (%)	Boys (%)	Girls (%)
*Income trajectory*				
Lowest	2.70 (2.52–2.89)	3.48 (3.29–3.68)	0.42 (0.36–0.50)	0.17 (0.13–0.23)
Second lowest	1.63 (1.50–1.76)	2.42 (2.25–2.59)	0.32 (0.27–0.38)	0.12 (0.09–0.15)
Downward	1.48 (1.26–1.74)	2.30 (2.07–2.56)	0.29 (0.20–0.41)	0.16 (0.11–0.25)
Middle	1.35 (1.21–1.50)	1.80 (1.66–1.94)	0.32 (0.27–0.39)	0.12 (0.09–0.17)
Second highest	0.86 (0.73–1.00)	1.41 (1.28–1.54)	0.25 (0.20–0.33)	0.08 (0.05–0.13)
Highest	2.10 (0.63–6.92)	1.35 (1.12–1.64)	0.18 (0.10–0.31)	0.21 (0.09–0.47)
All	1.71 (1.61–1.81)	2.31 (2.24–2.39)	0.32 (0.30–0.36)	0.13 (0.11–0.15)

## Discussion

In this register-based study including all individuals born in Finland in 1991–2005, we observed that low household income during childhood was associated with youth suicide attempts after considering parental factors including mental disorder, suicide, urbanicity, age, education and birth origin. Permanently low income appeared to be a more significant risk factor than a decrease in income. By contrast, the association between household income during childhood and suicide death was modest and attenuated completely when parental mental disorders were considered. Our findings were largely consistent using different income indicators. To the best of our knowledge, this is the first study to report the associations between income during childhood and both youth suicide attempts and deaths, within the same population.

Our results regarding low income in childhood as a risk factor for suicide attempts are mostly in line with previously reported findings from Denmark, Sweden and Finland [16, 33, 34]. A Danish study considered the income during childhood and concluded that the longer a child lived in poorer circumstances, the higher was the subsequent risk of hospital-treated self-harm among adolescents and young adults [16]. In a Swedish study on adolescents under 18 years of age, low parental income at age 12 was modestly associated with self-harm cases treated in outpatient services, but not with hospital-treated self-harm [33]. Some previous research has noted current low parental income as an independent risk factor for suicide attempts, with the risk appearing to be multiplicative when many co-existing adversities were present [15].

Childhood income modestly associated with the risk of suicide, and after cofactor adjustment this association was fully attenuated. Previous studies on suicide and childhood income have yielded conflicting results. A Danish register-based study found that low parental income increased the risk of suicide, although the association was attenuated after taking confounding factors into account [17]. In addition, receiving public assistance during childhood was observed to increase the risk of suicide when considered together with other adverse childhood experiences [35]. However, in one Norwegian study, suicide mortality was higher among those whose childhood income was higher, particularly among women [18]. A study from USA reported that insufficient income compared to the family’s needs positively associated with all-cause mortality among youths, whereas no link was observed with suicide deaths [36].

The contrasting results with previous studies could be explained by several contextual and methodological (e.g. definitions of the study variables, varying age groups) factors. Studies on income inequalities in youth suicidal behavior are few and largely based in high-income settings [37].

The gender distributions were consistent with previously observed patterns of suicides, which were more common among males whereas suicide attempts were more common among females [13]. This could be related to various gender-specific risk factors [13], as well as gendered and culturally prescribed scripts regarding suicide [38]. We observed no notable gender differences in income as a risk factor for suicide attempts and deaths. Household income showed no clear association with suicide mortality among girls, and the modest association among boys attenuated fully when cofactors were considered. Regarding suicide attempts, the associations were slightly stronger for girls, yet confidence intervals between the genders overlapped.

Income inequality plays a role in various health outcomes and mortality, which is often determined by structural factors that generate and perpetuate intergenerational cycles of disadvantage and poor health [39]. Although causal relationships cannot be established from this study, the possible mechanisms have been widely discussed in existing literature. Insufficient financial resources could cause stress and deprivation in living conditions, leading to less favorable health outcomes for the child, potentially also suicidal behavior. Possible pathways to increased suicide risk include accumulated adverse experiences, powerlessness and stigma, social exclusion, less healthy lifestyles and poor mental health [40]. On the other hand, poorer health of the parent or of the child could limit socioeconomic achievement. These mechanisms are often described as “social causation” and “social selection” hypotheses, which are not mutually exclusive [41]. While social causation emphasizes socioeconomic disadvantage and related environmental factors as antecedents of adverse health outcomes, social selection suggests that health-related vulnerabilities and traits can lead to lower socioeconomic status over time. When examining socioeconomic environment during childhood, the transgenerational and complex nature of these connections is present. Our findings support social causation, but also social selection as adjusting for parental factors including mental disorders attenuated the strength of the associations between household income and suicide attempts and deaths. This is consistent with previous knowledge indicating that parental mental disorders, as well as an individual’s own mental disorders, are risk factors for suicide and suicide attempts [30, 42].

Since more data exists on suicide attempts, and their prevalence is higher than that of completed suicides, the focus in research has often been on non-lethal suicidal behavior [11]. The rarity of suicide death as an outcome poses a challenge to conducting analyses, which may be manifested, for example, as insufficient statistical power. Evidence on outcome-risk factor specificity on different suicidal behaviors is hitherto limited [20].

The strengths of this study include the use of nationwide register data with comprehensive income records that permitted the longitudinal recording of the variables of interest and was free from participation and reporting bias. To the best of our knowledge, no previous register-based studies have examined suicide attempts and deaths separately in the same study cohort. To add, we conducted a multifaceted analysis using cumulative, trajectory, and age-specific income measures, which allows us to form a more complete picture how household income during childhood is associated with later suicide attempts and deaths. The study also encounters some important limitations that are common to register-based studies. National administrative registries are not originally collected for research purposes [43] which in this study, for example, limits the evaluation of suicidal intent of the study subjects. Suicide attempts and deaths in the administrative registries are more likely under- than overestimations [44]. There may be suicide attempts with serious intent that are not captured in the registers, if they have not led to medical contacts. However, suicide deaths are identified reliably based on the Finnish medicolegal death investigation standards, and few causes remain undetermined [45]. In line with previous studies [16, 25], confounders were measured at age 15 which may lead to conservative estimates. Moreover, our study was conducted in settings with a high level of social security and accessible health services by international standards, which limits the generalizability of the results.

In conclusion, this study contributes to the understanding of health disparities related to childhood socioeconomic inequality existing in the background of youth suicide attempts. However, the association of socioeconomic factors with youth suicide deaths is less evident. As childhood socioeconomic factors constitute a set of modifiable risk factors, they have the potential to affect various adverse health outcomes. Most of the existing national strategies don’t consider the reduction of inequalities in suicidal behavior [40]. Assuming social causation mechanism exists, interventions that improve people’s socioeconomic circumstances could potentially lower the prevalence of both mental disorders and suicide attempts [46]. However, more research is needed on different prevention strategies, including both universal, that aim to reduce risk factors in the whole populations, and selective interventions that improve the less advantaged people’s socioeconomic position [39]. Furthermore, our findings could have theoretical implications on suicidal behavior, since our results emphasize that dying from suicide is a rare and extreme event, and that the use of non-lethal suicidal behavior as a proxy for suicide should therefore be regarded with due caution.

## Supplementary Information

Below is the link to the electronic supplementary material.

Supplementary Material 1

## Data Availability

The data that support the findings of this study are available from the National Institute of Health and Welfare (www.thl.fi) and Statistics Finland (www.stat.fi). Restrictions apply to the availability of these data, which were used under license for this study. Inquiries about secure access to data should be directed to data permit authority Findata (findata.fi/en).
