# Supplementary material for: Household income during childhood and risk of youth suicide attempts and deaths: a register-based study from Finland
Source: Soc Psychiatry Psychiatr Epidemiol. 2026 Jun 15;61(11):2325–34. doi: 10.1007/s00127-026-03140-1 (PMC13694301; doi:10.1007/s00127-026-03140-1)
Supplement: Supplementary file 1 [file 127_2026_3140_MOESM1_ESM.docx]

Supplementary Figure 1. The associations of youth suicide attempts and death with household income quintiles at ages 5, 10, and 15.

*Results from stratified Cox regression models with minimal and additional adjustment. Minimally adjusted models were stratified with categorized birth year. The models examining suicides among boys and girls were stratified by year of birth and parental mental disorder by the given age (5, 10 or 15). Additionally adjusted models examining suicide attempts were stratified by year of birth as well as by parental age, parental education by the given age (5, 10 or 15), parental mental disorder by the given age (5, 10 or 15), parental suicide by the given age (5, 10 or 15), parental birth origin (in Finland or abroad), and urbanicity. Hazard ratios (HRs) and 95 % confidence intervals.*
